# A Neural Marker for Social Bias Toward In-group Accents

**DOI:** 10.1093/cercor/bhu282

**Published:** 2014-12-01

**Authors:** Patricia E.G. Bestelmeyer, Pascal Belin, D. Robert Ladd

**Affiliations:** 1 School of Psychology, Bangor University, Bangor, Gwynedd, UK; 2 Institute of Neuroscience and Psychology, University of Glasgow, Glasgow, UK; 3 International Laboratories for Brain, Music and Sound Research, Université de Montréal & McGill University, Montréal, Canada; 4 Institut des Neurosciences de La Timone, UMR 7289,CNRS & Aix-Marseille Université, Marseille, France; 5 School of Philosophy, Psychology and Language Sciences, University of Edinburgh, UK

**Keywords:** accent, fMRI, group membership, language

## Abstract

Accents provide information about the speaker's geographical, socio-economic, and ethnic background. Research in applied psychology and sociolinguistics suggests that we generally prefer our own accent to other varieties of our native language and attribute more positive traits to it. Despite the widespread influence of accents on social interactions, educational and work settings the neural underpinnings of this social bias toward our own accent and, what may drive this bias, are unexplored. We measured brain activity while participants from two different geographical backgrounds listened passively to 3 English accent types embedded in an adaptation design. Cerebral activity in several regions, including bilateral amygdalae, revealed a significant interaction between the participants' own accent and the accent they listened to: while repetition of own accents elicited an enhanced neural response, repetition of the other group's accent resulted in reduced responses classically associated with adaptation. Our findings suggest that increased social relevance of, or greater emotional sensitivity to in-group accents, may underlie the own-accent bias. Our results provide a neural marker for the bias associated with accents, and show, for the first time, that the neural response to speech is partly shaped by the geographical background of the listener.

## Introduction

Humans perceive their surroundings in terms of categories in which they compare the sensory information to several stored representations of objects, individuals, or social situations. Social categorization involves classifying individuals in terms of the groups they belong to (in-group) or do not belong to (out-group) and is a fundamental process in person perception (Bartlett 1932; Bruner 1957, 1958). Social theorists have argued that, in addition to our personal identities, these social categories are so important to us that our identity is partially based on these group memberships (e.g., Tajfel and Turner 1979; Turner et al. 1987). Thus the groups we belong to shape our attitudes determine the language we speak and which accent we have. The purpose of the formation of these categories is thought to simplify the overwhelming environment the perceiver is confronted with (Brewer 1988; Fiske and Neuberg 1990), but this computational reductionism comes with costs. Social identity theory predicts that group membership causes an enhancement and favoritism of the in-group at the expense of the out-group. This theory has found support in numerous experimental studies which have reported that the mere perception of belonging, or even just the awareness of the presence of 2 distinct groups, is sufficient for a bias toward or favoring of the in-group (Tajfel et al. 1971; Doise et al. 1972; Billig and Tajfel 1973; Tajfel and Billig 1974; Turner 1975). Later studies have suggested that this bias occurs without conscious awareness and involves positive affect toward the in-group (Dovidio and Gaertner 1993) even when the in-group is novel or based on arbitrary categorization (Otten and Moskowitz 2000).

Many aspects of group identity are conveyed by a person's accent, including geographical, socio-economic, and ethnic background (Labov 2006). Listeners are highly sensitive to these phonetic, phonological, and prosodic variations in speech and often use the information provided by accents to make important social judgments about the speaker such as the speaker's personality (Dailey et al. 2005). In line with social identity theory, individuals typically judge their own accent or the accent most similar to their own as more favorable (Hurt and Weaver 1972; Mulac et al. 1974; Ryan and Sebastian 1980; Edwards 1982; Coupland and Bishop 2007) and trustworthy (Lev-Ari and Keysar 2010). An own-race bias for voices has recently been demonstrated in White and Black Americans (Perrachione et al. 2010). In this study participants were relatively accurate at categorizing the race of the speakers. Both races displayed an advantage of identifying their own racial group, the hallmark of an own-group bias effect. However, this accuracy was largely driven by the dialect of the speakers rather than differences in vocal structure of the 2 races and as such Perrachione et al. (2010) provide further evidence for the existence of an own-accent bias. Cohen (2012) proposes that accents have evolved to furnish the “honest signal” of group membership needed to drive the growth of non-kin cooperation in human evolutionary history.

Applied research supports the impact of this own-accent bias. In higher education, for example, North American students rated North American teachers more favorably and recalled more information from their lessons than from teachers who spoke British or Malaysian-accented English (Gill 1994). Similarly, a recent study on “ear witness” memory reported an interaction between witness accent and offender's accent in that Scottish and English ear witnesses were less confident in their judgment and more prone to confuse offenders who spoke in a different accent to their own (Stevenage et al., (2012); see also Philippon et al. (2007) for a similar result with familiar versus unfamiliar accented speech). Individuals with out-group accents may sound more alike and may therefore be more easily confused (Williams et al. 1999). Developmental research comparing native and foreign-accented speech shows that this bias emerges early in life and cannot be entirely explained by intelligibility of foreign-accented speech: by 5 years of age children prefer native to foreign-accented speakers as friends even when they comprehend both speakers (Hirschfeld and Gelman 1997; Kinzler et al. 2007).

The neuroscience of accent perception has only recently received attention in a study which investigated the neural substrates of accent processing of standard Dutch and an artificial, novel variation of Dutch (Adank et al. 2012). It revealed that bilateral mid and superior temporal gyri (STG), planum temporale, as well as left inferior frontal gyrus (IFG) are involved in processing accents. Some of the reported activations overlapped with regions that also respond preferentially to vocal compared with non-vocal sounds known as the temporal voice areas (TVAs) (Belin and Zatorre 2000; Grandjean et al. 2005; Lewis et al. 2009; Bestelmeyer et al. 2011). This study probed accent perception using a tool commonly used in cognitive science known as adaptation. Adaptation, also referred to as the “psychologist's microelectrode” (Frisby 1980), usually results in a reduction of neural activity as a consequence of stimulus repetition and has widely been used to reveal neural populations tuned to respond to specific stimulus attributes [see Grill-Spector et al. (2006) for a review]. While the underlying mechanisms of adaptation are still debated there is agreement that the amount of repetition suppression (or decrease in neural activity) is related to the ability of the neural population to discriminate repeating stimuli (Grill-Spector et al. 2006). In other words, the more similar two repeating stimuli are perceived to be the greater the amount of repetition suppression. Conversely, it has been shown that if repeated stimuli are of greater social relevance or are more attended to, repetition suppression is less pronounced, and may even result in increased neural activity or repetition enhancement [Kouider et al. 2007; Nakamura et al. 2007; and see Segaert et al. (2013) for a review]. A neural marker for social bias toward in-group accents should be evident in an interaction between speaker and listener accent. There are at least 2 possible hypotheses regarding the neural substrates and also the direction this interaction may involve. These are outlined below.

One possible explanation for the positive bias toward own-accented speakers may be driven by an emotional reaction toward them. Neuroscientific research on vocal emotion and affective prosody converges on the involvement of largely right-lateralized activation of the mid and superior temporal gyri (Mitchell et al. 2003; Grandjean et al. 2005; Wildgruber et al. 2005; Ethofer et al. 2006; 2009; Leitman et al. 2010), insulae (Klasen et al. 2011; Frühholz and Grandjean 2012) and IFG (Ethofer et al. 2012; Frühholz and Grandjean 2012) as well as subcortical structures such as the basal ganglia (Pell and Leonard 2003) and amygdalae (Phillips et al. 1998; Morris et al. 1999; Sander and Scheich 2001; Sander et al. 2003, 2005; Fecteau et al. 2007; Wiethoff et al. 2009; Leitman et al. 2010; Klasen et al. 2011; Ethofer et al. 2012; Frühholz and Grandjean 2013). Though the amygdalae used to be seen as the center for affective information processing we now know that these structures much more broadly deal with social cognition and particularly social relevance (e.g., Schirmer et al. 2008). Hence these structures have been dubbed “relevance detectors” (Sander et al. 2003). Areas responding to vocal emotion and, more generally, social relevance, would therefore be likely candidates for coding group membership based on accents with reduced repetition suppression to own compared with other accents.

An alternative, but not mutually exclusive, hypothesis comes from theories developed in cognitive psychology and linguistics based on the notion of stored prototypes which aid categorization (Rosch 1973; Valentine 1991). This may be the reason for faster adjustment or normalization to familiar compared with foreign-accented sentences [Floccia et al. 2006, although this pattern was not evident for single words; see also Evans and Iverson (2004) and Cristia et al. (2012) for a comprehensive review]. In this view, the own-accent bias could be due simply to a familiar internal representation or prototype of our own accent; any accent that deviates from this acoustic prototype would be classed as distinct or “other.” It is also consistent with recent research showing that voices located further away from the average voice (or prototype) are judged as more distinctive and less attractive (Bruckert et al. 2010; Bestelmeyer et al. 2012). This research has found that increasing vocal distinctiveness correlates positively with fMRI signal in bilateral TVAs. If the existence of familiar acoustic prototypes is the basis of the own-accent bias, we would predict the neural correlates of the interaction between listener and speaker accent in specialized auditory regions. Importantly, given the findings on faster normalization to familiar compared with unfamiliar accents we would expect the *reverse* interaction to the one expected if the bias was driven by social relevance or emotion. In other words, we would predict increased repetition suppression to own compared with other accents.

The aim of the present study was to identify a neural marker for the bias toward the accent of the in-group, and to discriminate between the two possible explanations outlined—affective processing versus prototype representation—to account for the own-accent bias. We used functional magnetic resonance imaging (fMRI) to measure blood oxygenation level-dependent (BOLD) signal which is an indirect index of neuronal activity. We scanned 2 groups of participants naïve to the purpose of the study: one from Scotland and the other from the South of England, while they passively listened to 3 different native English accents (Southern English, Scottish, and American) expressed in short utterances of numbers. A strong test of the own-accent bias requires a significant interaction between the accent of the listener and the accent of the speaker. We therefore specifically predicted an interaction with 2 alternative patterns of activations and explanations. The first possible hypothesis leads us to expect this interaction in areas which are involved in vocal affect perception and relevance detection such as superior and midtemporal gyrus/sulcus (STG/STS), IFG, and amygdala. If the own-accent bias is better explained by the notion of an internal prototype, against which all other accents are evaluated, we would expect the interaction to be exclusive to areas that encode acoustic differences such as Heschl's gyri and secondary auditory cortex.

## Method

### Participants

Twenty Scottish volunteers from the undergraduate and postgraduate community of the University of Glasgow took part (11 females, mean age = 23.45, standard deviation (SD) = 3.62). Twenty Southern English participants from the undergraduate and postgraduate communities of the Universities of Glasgow and Edinburgh took part (8 females, mean age = 18.80, SD = 1.44). The Scottish participants had all lived their whole life in Scotland. Southern English participants had all lived their whole life in the South of England and most had been in Scotland for no longer than 4 months. All participants spoke with an accent that was typical of their geographical origin. Participants were naïve to the purpose of the study, reported normal hearing and were reimbursed £12 for their time (£6/h) plus £20 if they had to travel from Edinburgh. Informed consent was obtained from all individuals and the study protocol was approved by the local ethics committee.

### Stimuli and Paradigms

A total of 14 female native southern English, Scottish, and General American speakers were selected and recorded by an experienced phonetician in a professional-quality studio at the University of Edinburgh. Speakers were recorded uttering 8 different 4-digit numbers. Stimuli were tested for recognizability in a brief pilot study of 10 naïve native British English participants (who did not participate in the MRI study). In this pilot study, each stimulus was presented twice. We selected 3 speakers of each accent group and each speaker uttered 3 types of number sequences (“1-4-2-9”, “2-4-5-8,” and “9-8-3-4”). The selection of these speakers was based on high categorization accuracy (∼90%) on a self-paced 3-alternative forced choice task.

General American accents served as filler trials or “null events” and were modelled at first- and second-level analyses. Importantly, these trials helped ensure that participants would remain unaware of the purpose of the study. Stimuli were normalized in energy (root mean square). The duration of the 9 speech samples ranged from 1.18 to 1.86 s. Post hoc tests revealed that speech recordings of the 3 accent types did not differ significantly in F0, f1, f2, HNR, and duration.

We employed a continuous carry-over design (Aguirre 2007) to measure the effects of one stimulus upon the next using a first-order serially balanced sequence of stimuli known as type-1-index-1 (Nonyane and Theobald 2007). In this sequence each stimulus is preceded and followed by every other stimulus an equal number of times and was defined by 9 items (3 accents × 3 number sequences). Each run therefore consisted of 82 stimuli and was repeated 9 times. Each run was divided by 20 s of silence. Stimuli were presented binaurally using the electrostatic NNL headphone system (NordicNeuroLab, Inc.) at an intensity of 80 dB SPL(C). Participants were asked to keep their eyes closed, listen passively to the numbers, and press a button at the beginning and end of each run. All 9 runs were acquired in one session. No participant missed more than 2 button presses.

During the voice localizer participants were instructed to listen passively to 10 s blocks of either vocal sounds (*n* = 21) or non-vocal sounds (*n* = 21) interspersed with silent blocks (*n* = 21) presented in a pseudo-randomized order. Each block started with 2 s of silence followed by 8 s of different stimuli of the same category (see Belin and Zatorre (2000). Vocal sounds consist of brief segments of speech (e.g., syllables, words, and sentences in foreign languages) and non-speech (e.g., laughs, sighs, coughs) vocalizations. Non-vocal sounds consist of industrial and environmental sounds.

After scanning, participants were asked to categorize all voices in terms of their accent to make sure participants were able to recognize each accent. Each of the 3 digit strings of each of the 9 speakers were presented 3 times via Beyerdynamic headphones in a quiet room at an intensity of 70 dB SPL (C). Participants had to press one of the 3 buttons in response to each accent (a total of 81 trials). We used the Psychtoolbox3 (Brainard 1997; Pelli 1997) for stimulus presentation in the fMRI and behavioral task based on MatlabR2007b (Mathworks, Inc.).

### Image Acquisition and Analysis

All MRI scans were acquired in a 3.0 T Siemens Tim Trio scanner using a 12-channel head coil. Both *T*_2_*-weighted functional scans were acquired using an echo-planar imaging (EPI) sequence (32 axial slices; voxel size: 3 × 3 × 3 mm^3^; 70 × 70 matrix; flip angle: 77°; FOV = 210; 0.3 mm gap between slices) and an interleaved ascending order. The experimental run consisted of one fast event-related scan (TR = 2 s, TE = 30 ms; 828 volumes; 28 min). The voice localizer (TR = 2 s; TE = 30 ms; 310 volumes; 10 min) allows reliable identification of the temporal voice areas (TVAs) using the vocal versus non-vocal contrast. In both functional scans the sounds were superimposed on scanner noise. Whole brain *T*_1_-weighted anatomical scans were performed using fast gradient echo known as T_1_ “Magnetization Prepared Rapid Gradient Echo” (MPRAGE; 192 axial slices; voxel size: 1 × 1 × 1 mm^3^; 256 × 256 matrix) performed at the end of the experimental session.

All MRI data were analyzed using SPM8 (Wellcome Department of Imaging Neuroscience, 1994–2007; http://www.fil.ion.ucl.ac.uk/spm). First, anatomical scans were AC-PC aligned with the re-orientation applied to all EPI scans done in the same session. Pre-processing of functional scans consisted of corrections for head motion (spatial realignment; trilinear interpolation) and scans were realigned to the first volume of the last functional scan (i.e., the volume closest to the anatomical scan). Functional runs were then co-registered to their corresponding individual anatomical scans. Functional (3 mm isotropic voxels) and anatomical (1 mm isotropic voxels) data were transformed to Montreal Neurological Institute (MNI) space after segmentation of the anatomical scans. Normalized data were spatially smoothed by applying a Gaussian kernel of 8 mm full width at half maximum.

The experimental run was analyzed using parametric modulations to keep it consistent with previous literature on carry-over designs (e.g., Aguirre 2007; note that the data could also be analyzed categorically—this equivalent analysis yields very similar results and is detailed in Supplementary material section). We coded each accent separately with 0 corresponding to “no carry-over” (no accent repetition) and 1 corresponding to “carry-over” (accent repetition) trials. The analysis therefore consisted of 3 main parametric modulators of interest: carry-over effects for the 1) Southern English accent, 2) Scottish accent, and 3) American accent. Typically, in a parametric modulation analysis in SPM8, additional regressors are orthogonalized from left to right in a given matrix so that the shared variance of one regressor is removed from the next (Büchel et al. 1998). However, we disabled this feature in SPM8 so that the order in which the parametric modulators were entered did not affect the results and allowed us to investigate the unique variance of each regressor (see http://imaging.mrc-cbu.cam.ac.uk/imaging/ParametricModulations for details).

We checked that the groups did not differ in their basic voice cognition abilities by assessing any group differences in the TVAs using a two-sample *t*-test. We tested for the predicted interaction by computing a flexible factorial design ANOVA. This design assessed the variance of several factors: 1) subjects, 2) groups (2 levels: southern English, Scottish participants), and 3) conditions (3 levels: Southern English, Scottish, and American accents). We used this design to examine increased activations to repetitions of a groups' own accent compared with the accent of the other participant group as well as the reverse interaction (note we modeled the American accent but did not include it in the interaction contrast).

Reported results are from whole brain analyses. For the voice localizer block design we used a conservative threshold of *P* < 0.05, family-wise error (FWE) corrected at the voxel level for the whole brain. For the fast event-related design, statistical significance was assessed at FWE corrected at the cluster level with a threshold of *P* < 0.05 (corresponding to a cluster size of at least 50 voxels). Results are illustrated on an average anatomical scan using MRIcron (Rorden et al. 2007) at a height threshold of *P* < 0.001 (uncorrected) and an extent threshold of 50 voxels to illustrate all significant maxima (Fig. 2*A*–*D*). Illustration of the voice localizer (Fig. 2*E*) is set at *P* < 0.05 (FWE corrected at voxel level). To illustrate parameter estimates in Figure 2 we used SPM8's built-in function (spm_graph.m) to extract the beta estimates at the 4 peak maxima within a sphere (radius of 3 mm). Anatomical cluster location was assessed with xjview (8.1; http://www.alivelearn.net/xjview) and cross checked with Duvernoy's brain atlas (Duvernoy 1999) to ensure accuracy.

## Results

### Behavioral Results

**Figure 1.**
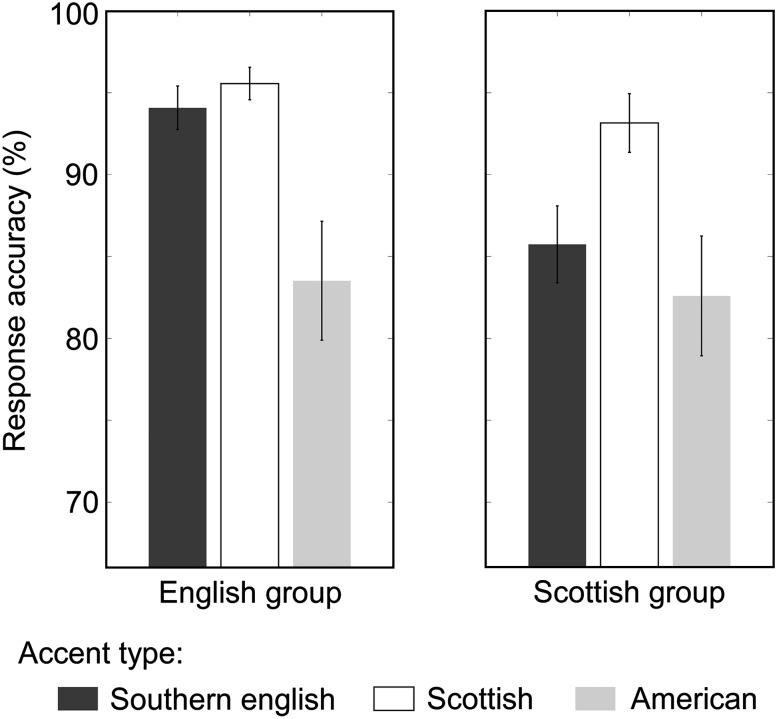
Bar graphs represent response accuracy (%) for each of the two participant groups for the 3 different accents. Error bars represent standard error of the mean.

Results of a behavioral test evaluating the ability of listeners to identify the 3 accents correctly are summarized in Figure 1. Both groups performed significantly better than chance level for all accents (all *t* > 13.5, *P* < 0.0001). We carried out a mixed-design ANOVA to test for the predicted interaction between participant group and accent type (Southern English, Scottish) which was significant (*F*_1, 37_ = 4.31, *P* = 0.045, *pη*^2^ = 0.10). An independent samples *t*-test revealed that this interaction was driven by the Scottish group being significantly better at recognizing their own accent compared with the Southern English accent (*t*_(3,7)_ = −3.13, *P* = 0.003). The English group was equally good at recognizing either accent (*t*_(3,7)_ = −1.33, *P* = 0.19) possibly because this group had already lived in Scotland for several months at the time of testing.

### Neuroimaging Results

Contrasts of each accent versus the silent baseline showed the classic pattern of bilateral auditory activation in both the Scottish and English listeners. Similarly, all participants had normal bilateral activations in response to vocal compared with non-vocal sounds (e.g., bells) as assessed with the voice localizer block design (Belin and Zatorre 2000) with the expected group maximum in right STG/STS (Scottish: 54 -19 1, cluster size (*k*) = 560, *T*-value = 13.14; English: 63 -22 -2, *k* = 438, *T*-value = 17.47) and a second cluster in the left STS/STG (Scottish: -63 -16 1, *k* = 443, *T*-value = 12.90; English: -60 -16 4, *k* = 445, *T*-value = 14.71). A 2-sample *t*-test revealed no significant differences (*P* > 0.05; corrected at cluster level) between groups in terms of their neural activations to voices compared with environmental sounds.

**Figure 2.**
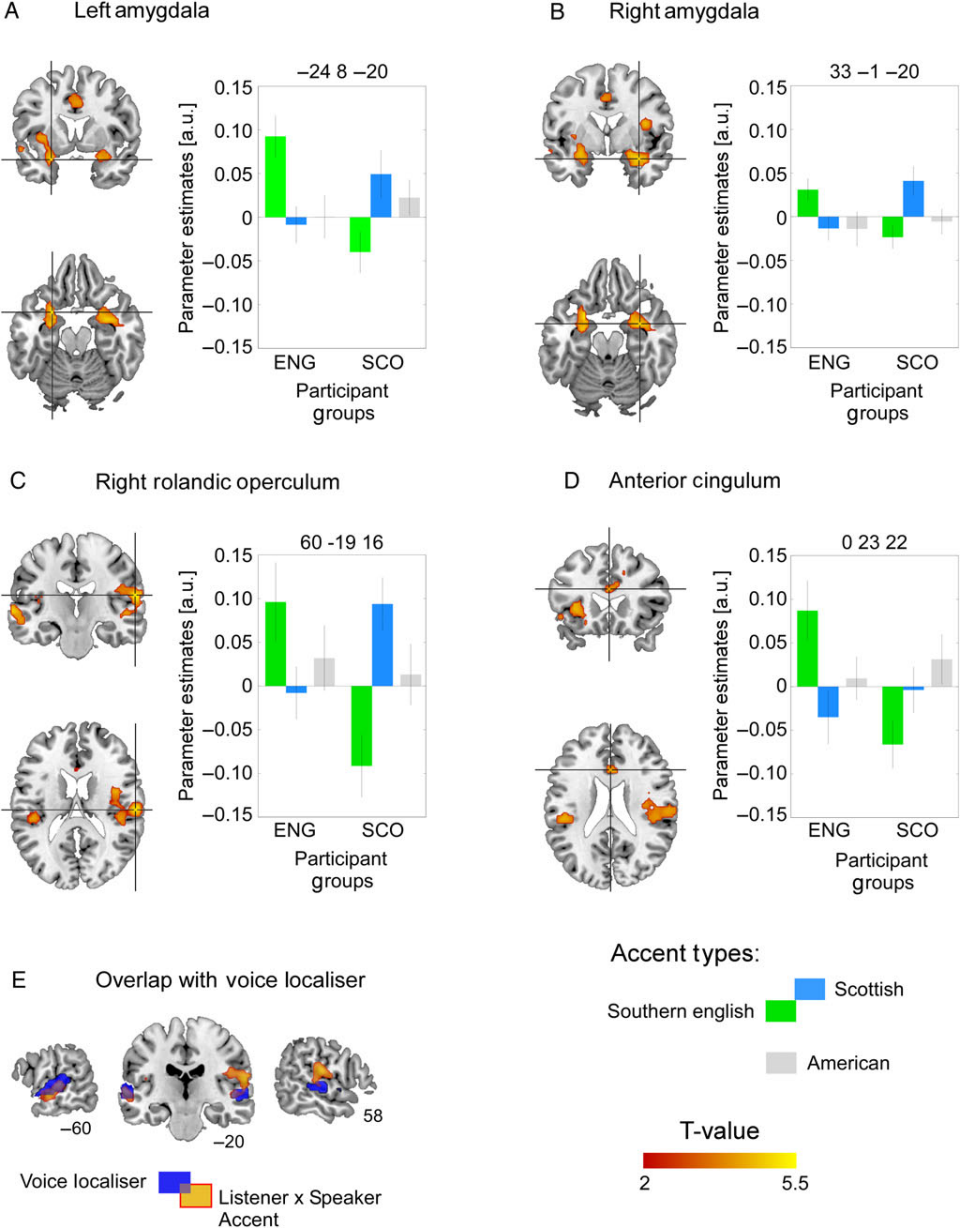
Overlay of significant interaction between participant group (Scottish, Southern English) and accent type of the speakers (Scottish, Southern English) in (*A*) left amygdala, (*B*) right amygdala, (*C*) right rolandic operculum, and (*D*) anterior cingulum. Bar graphs represent the parameter estimates in the peak voxels of each significant cluster and clearly indicate the interaction between the accent type of the speakers (green: Southern English; blue: Scottish; gray: American for completion) and the accent of the listeners. Error bars represent standard error of the mean. (*E*) Significant interaction between participant group (listeners) and accent type (speakers) overlaid on voice-sensitive areas of cortex (dark blue).

The predicted interaction between the geographical background of the participant and the accent type the participant listened to is illustrated in Figure 2*A*–*D* with the parameter estimates to the American accent shown in gray for completeness. Participants of both groups showed repetition enhancement to their own accent but decreased neural response (or repetition suppression) to the repetition of the other group's accent. Significant clusters emerged in left amygdala (Fig. 2*A*; peak maximum: −24 8 −20 involving left midtemporal gyrus with *k* = 607 and *T*-value = 4.84), right amygdala (Fig. 2(*B*); peak maximum: 33 −1 −20 involving right midtemporal gyrus with *k* = 190 and *T*-value = 4.58), right rolandic operculum (Fig. 2*C*; peak maximum: 60 −19 16; involving insula and STG with *k* = 395 and *T*-value = 4.90) and anterior cingulum (Fig. 2(*D*); peak maximum: 0 23 22 with *k* = 217 and *T*-value = 3.88). In these regions, consecutive stimuli spoken with the participant's regional accent led to activity increases, while repetition of the out-group accent led to the classic repetition-induced activity decreases. No brain region showed the reverse interaction, that is, we found no areas that responded more to the accent of the out-group. Figure 2*E* shows the overlap between the voice-sensitive regions (as revealed by the voice localizer) and the activation from the predicted interaction.

## Discussion

This study aimed at identifying a neural marker for the social bias toward own accents. While this bias has been reported on in various areas of psychology, education, marketing, and sociolinguistics, its neural underpinnings were unexplored. Our neuroimaging data are the first to provide a neural signature for the “own-accent bias” evidenced as a significant interaction between the accent of the participant and the accent of the speakers. Specifically, repetitions of the participant's own accent were associated with increased activation in bilateral amygdalae, right rolandic operculum, and anterior cingulum, while repetitions of the other group's accent showed decreased activations in these regions. In contrast, there were no significant findings of the reverse interaction, that is, of stronger activation to out-group accents. Our results are the first to suggest a neural signature for social group membership conveyed simply by means of regional variations in pronunciation of the same language.

While previous behavioral work documents the existence of this bias, it was unclear what might drive it. We suggested 2 hypothetical accounts of the underlying neural architecture based on previous behavioral and neuroimaging literature. First, the own-accent bias may be driven by an emotional reaction toward or social relevance detection of our own group which would suggest activation of areas sensitive to auditory affective content such as the amygdalae and STS/STG (e.g., Sander et al. 2005; Ethofer et al. 2012; Witteman et al. 2012; Bestelmeyer et al. 2014). Second, we thought it was possible that the own accent bias is a result of accents being processed in terms of a prototype against which individuating information such as accents are coded which would imply involvement of regions sensitive to acoustic differences [Rosch 1973; see also Leopold et al. (2001) for a similar notion in the face literature] such as Heschl's gyri and secondary auditory cortex. Our data seem to rule out the latter account. We found no significant interaction and, importantly, no greater adaptation to in-group accents in bilateral primary and secondary auditory cortices, which is what would be predicted had the prototype account been supported. Instead we found a clear interaction with generally increased fMRI signal to own accents and decreased signal to the out-group accent in areas typically associated with auditory affective content (see Fig. 2).

Our results are in line with neuroimaging studies in a related research field of the visual domain. One recent study investigated the effect of implicit racial bias in own versus other-race faces (Van Bavel et al. 2008). Participants viewing novel in-group compared with out-group faces showed greater activity in left amygdala and left orbitofrontal cortex [see also Volz et al. (2009) for similar results], as well as in areas typically seen in neuroimaging studies of face perception (i.e., fusiform gyri). Amygdala activity in this context may stem from greater affective salience or relevance of in-group compared with out-group faces (Whalen 1998; Anderson and Phelps 2001; Vuilleumier 2005).

Voice-sensitive cortex has also been shown to respond more to more behaviorally relevant stimuli. More specifically, when Ethofer et al. (2007) presented female and male listeners with erotic prosody spoken by female and male actors voice-sensitive cortex (particularly right superior midtemporal gyrus) responded more to the voices of the opposite sex. Thus voice-sensitive cortex, with which our own-accent interaction partially overlaps, also shows sensitivity to voices that have high behavioral relevance for the listener. Another study which is relevant for the interpretation of our findings contrasted pleasant with unpleasant musical excerpts. This research revealed activation patterns in bilateral IFG, ventral striata, Heschl's gyri, and rolandic opercula as well as subcortical structures such as the amygdala and hippocampus (Koelsch et al. 2006). Heschl's gyri and rolandic opercula are also areas which respond to vocal affect (Ethofer et al. 2012). Our activation patterns, as revealed by the interaction between listener and speaker accent, show remarkable resemblance to activations in response to pleasant music, vocal affect, and stimuli with increased behavioral relevance to the participant. Taken together our results support an emotional account of the own-accent bias.

The present study used an adaptation paradigm to investigate a neural marker for the bias toward own accents. Neurally, fMRI adaptation to a specific stimulus is typically accompanied by a decrease in the hemodynamic response (Grill-Spector et al. 2006). The rationale behind adaptation studies is that repetition of the same stimulus type results in response suppression which reveals neural populations that are tuned to the processing of a specific stimulus attribute, that is, repetition suppression reveals functional specificity of neural populations. As predicted by the adaptation framework we should have seen varying degrees of repetition suppression to both accents. Instead we observed reduced activation only to the accent of the out-group but not to the accent of the in-group. Several studies have observed repetition enhancement in adaptation designs under various conditions and stimulus types but explanations underlying this phenomenon are scarce [e.g., Vuilleumier et al. 2005; Kouider et al. 2007; Turk-Browne et al. 2007; Müller et al. 2013; and see Henson (2003) and Segaert et al. (2013) for a review]. A tentative explanation of our result is that expertise in the accent of the out-group is limited while sensitivity to (e.g., precise origin), and social relevance of, the in-group accent is enhanced, thereby disrupting repetition suppression. This explanation of repetition enhancement to own accents is in line with a previous study showing this neural pattern to objects with learned behavioral relevance (Desimone 1996).

The activation to the repetition of the in-group accent was typically greater than to both out-group accents. This pattern was particularly pronounced for the 2 British accents. The interaction between participant background and accent of the speaker they listened to may be due to the long-standing and deep rivalry between England and Scotland, which were debating the separation of a political union in the Scottish independence referendum in September 2014. North America has not played a prominent role in these current and historical political debates and thus attitudes of both British groups toward Americans may be less intense. While this is speculative, environmental effects such as culture are known to affect neural processing (Goh et al. 2007, 2010) and it is therefore conceivable that these historical rivalries shape language attitudes and thereby the perception of accented speakers.

Many important questions remain with regards to linking behavior to the neural pattern we observe for a firmer interpretation of the etiology of a neural marker for group membership. As such we should be able to tie the size of this repetition enhancement to own-accents with measures of national identity and attitudes. Similarly, it remains to be determined whether the size of the observed effect relates to actual stereotyping behavior. Hence it would be encouraging to see years of residency and, more importantly, social integration into the out-group's environment, reduce the size of the repetition suppression effect to the out-group accent.

## Conclusion

Our study aimed at quantifying and providing neural support for the own-accent bias observed in previous behavioral reports. We found a significant interaction for repetition trials between the accent type of the listener and the accent type of the speaker in bilateral amygdalae, right rolandic operculum and anterior cingulum. In these regions we observed reduced activity to the out-group accent but repetition enhancement to the participant's own accent. We cautiously interpret this finding in terms of increased sensitivity to and perceived relevance of own accents compared with out-group accents. Our results also indicate that the neural response to accents depends on, and is shaped by, the listener's own linguistic background. Our results are the first report of a neural signature for group membership based on phonetic variations of the same language.

## Funding

This work was supported by the Economic and Social Research Council/Medical Research Council grant (RES-060-25-0010). Funding to pay the Open Access publication charges for this article was provided by the RCUK block grant.

## References

[BHU282C1] Adank P Noordzij ML Hagoort P 2012 The role of planum temporale in processing accent variation in spoken language comprehension. Hum Brain Mapp. 33:360–372. 21391272 10.1002/hbm.21218 PMC6870325

[BHU282C2] Aguirre GK 2007 Continuous carry-over designs for fMRI. Neuroimage. 35:1480–1494. 17376705 10.1016/j.neuroimage.2007.02.005 PMC2147064

[BHU282C3] Anderson AK Phelps EA 2001 Lesions of the human amygdala impair enhanced perception of emotionally salient events. Nature. 411:305–309. 11357132 10.1038/35077083

[BHU282C4] Bartlett FC 1932 Remebering: a study in experimental and social psychology. New York: Cambridge University Press.

[BHU282C5] Belin P Zatorre RJ 2000 ‘What’, ‘where’ and ‘how’ in auditory cortex. Nat Neurosci. 3:965–966. 11017161 10.1038/79890

[BHU282C6] Bestelmeyer PEG Belin P Grosbras M-H 2011 Right temporal TMS impairs voice detection. Curr Biol. 21:R838–R839. 22032183 10.1016/j.cub.2011.08.046

[BHU282C7] Bestelmeyer PEG Latinus M Bruckert L Crabbe F Belin P 2012 Implicitly perceived vocal attractiveness modulates prefrontal cortex activity. Cereb Cortex. 22:1263–1270. 21828348 10.1093/cercor/bhr204

[BHU282C8] Bestelmeyer PEG Maurage P Rouger J Latinus M Belin P 2014 Adaptation to vocal expressions reveals multi-step perception of auditory emotion. J Neurosci. 34:8098–8105. 24920615 10.1523/JNEUROSCI.4820-13.2014 PMC4051968

[BHU282C9] Billig M Tajfel H 1973 Social categorization and similarity in intergroup behaviour. Eur J Soc Psychol. 3:27–52.

[BHU282C10] Brainard DH 1997 The Psychophysics Toolbox. Spat Vis. 10:433–436. 9176952

[BHU282C11] Brewer MB, editor. 1988 A dual process model of impression formation. Hillsdale, NJ: Lawrence Erlbaum.

[BHU282C12] Bruckert L Bestelmeyer P Latinus M Rouger J Charest I Rousselet GA Kawahara H Belin P 2010 Vocal attractiveness increases by averaging. Curr Biol. 20:116–120. 20129047 10.1016/j.cub.2009.11.034

[BHU282C13] Bruner JS 1957 On perceptual readiness. Psychol Rev. 64:123–152. 13420288 10.1037/h0043805

[BHU282C14] Bruner JS 1958 Social psychology and perception. In: Maccoby EE Newcomb TM Hartley EL, editors. Readings in social psychology, 3rd ed. New York: Henry Holt and Company p. 85–94.

[BHU282C15] Büchel C Holmes AP Rees G Friston KJ 1998 Characterizing stimulus–response functions using nonlinear regressors in parametric fMRI experiments. Neuroimage. 8:140–148. 9740757 10.1006/nimg.1998.0351

[BHU282C16] Cohen E 2012 The evolution of tag-based cooperation in humans: the case for accent. Curr Anthropol. 53:588–616.

[BHU282C17] Coupland N Bishop H 2007 Ideologised values for British accents. J Socioling. 11:74–93.

[BHU282C18] Cristia A Seidl A Vaughn C Schmale R Bradlow A Floccia C 2012 Linguistic processing of accented speech across the lifespan. Front Psychol. 3:479–479. 23162513 10.3389/fpsyg.2012.00479 PMC3492798

[BHU282C19] Dailey R Giles H Jansma L 2005 Language attitudes in an Anglo-Hispanic context: the role of the linguistic landscape. Lang Commun. 25:27–38.

[BHU282C20] Desimone R 1996 Neural mechanisms for visual memory and their role in attention. Proc Natl Acad Sci USA. 93:13494–13499. 8942962 10.1073/pnas.93.24.13494 PMC33636

[BHU282C21] Doise W Csepeli G Dann HD Gouge C Larsen K Ostell A 1972 Experimental investigation into formation of intergroup representations. Eur J Soc Psychol. 2:202–204.

[BHU282C22] Dovidio JF Gaertner SL 1993 Stereotypes and evaluative intergroup bias. In: Mackie DM Hamilton DL, editos. Affect, cognition, and stereotyping, San Diego: Academic Press p. 167–193.

[BHU282C23] Duvernoy HM 1999 The human brain: surface, blood supply, and three-dimensional sectional anatomy. Wien: Springer.

[BHU282C24] Edwards JR 1982 Language attitudes and their implications among English speakers. In: Ryan EB Giles H, editors. Attitudes towards language variation. London: Edward Arnold p. 20–33.

[BHU282C25] Ethofer T Anders S Wiethoff S Erb M Herbert C Saur R Grodd W Wildgruber D 2006 Effects of prosodic emotional intensity on activation of associative auditory cortex. Neuroreport. 17:249–253. 16462592 10.1097/01.wnr.0000199466.32036.5d

[BHU282C26] Ethofer T Bretscher J Gschwind M Kreifelts B Wildgruber D Vuilleumier P 2012 Emotional voice areas: anatomic location, functional properties, and structural connections revealed by combined fMRI/DTI. Cereb Cortex. 22:191–200. 21625012 10.1093/cercor/bhr113

[BHU282C27] Ethofer T De Ville DV Scherer K Vuilleumier P 2009 Decoding of emotional information in voice-sensitive cortices. Curr Biol. 19:1028–1033. 19446457 10.1016/j.cub.2009.04.054

[BHU282C89] Ethofer T Wiethoff S Anders A Kreifelts B Grodd W Wildgruber D 2007 The voices of seduction: cross-gender effects in processing of erotic prosody. SCAN. 2:334–337. 18985138 10.1093/scan/nsm028 PMC2566759

[BHU282C28] Evans BG Iverson P 2004 Vowel normalization for accent: an investigation of best exemplar locations in northern and southern British English sentences. J Acoust Soc Am. 115:352–361. 14759027 10.1121/1.1635413

[BHU282C29] Fecteau S Belin P Joanette Y Armony JL 2007 Amygdala responses to nonlinguistic emotional vocalizations. Neuroimage. 36:480–487. 17442593 10.1016/j.neuroimage.2007.02.043

[BHU282C30] Fiske ST Neuberg SL 1990 A continuum of impression formation, from category based to individuating processes: Influence of information and motivation on attention and interpretation. San Diego, CA: Academic Press.

[BHU282C31] Floccia C Goslin J Girard F Konopczynski G 2006 Does a regional accent perturb speech processing? J Exp Psychol Human. 32:1276–1293. 10.1037/0096-1523.32.5.1276 17002537

[BHU282C32] Frisby JP 1980 Seeing: illusion, brain and mind. Oxford: Oxford Press.

[BHU282C33] Frühholz S Grandjean D 2012 Towards a fronto-temporal neural network for the decoding of angry vocal expressions. Neuroimage. 62:1658–1666. 22721630 10.1016/j.neuroimage.2012.06.015

[BHU282C34] Frühholz S Grandjean D 2013 Amygdala subregions differentially respond and rapidly adapt to threatening voices. Cortex. 49:139401403. 10.1016/j.cortex.2012.08.003 22938844

[BHU282C35] Gill MM 1994 Accent and stereotypes: their effect on perceptions of teachers and lecture comprehension. J Appl Commun Res. 22:348–361.

[BHU282C36] Goh JO Chee MW Tan JC Venkatraman V Hebrank A Leshikar ED Jenkins L Sutton BP Gutchess AH Park DC 2007 Age and culture modulate object processing and object-scene binding in the ventral visual area. Cogn Affect Behav Neurosci. 7:44–52. 17598734 10.3758/cabn.7.1.44

[BHU282C37] Goh JO Leshikar ED Sutton BP Tan JC Sim SKY Hebrank AC Park DC 2010 Culture differences in neural processing of faces and houses in the ventral visual cortex. Soc Cogn Affect Neurosci. 5:227–235. 20558408 10.1093/scan/nsq060 PMC2894673

[BHU282C38] Grandjean D Sander D Pourtois G Schwartz S Seghier ML Scherer KR Vuilleumier P 2005 The voices of wrath: brain responses to angry prosody in meaningless speech. Nat Neurosci. 8:145–146. 15665880 10.1038/nn1392

[BHU282C39] Grill-Spector K Henson R Martin A 2006 Repetition and the brain: neural models of stimulus-specific effects. Trends Cogn Sci. 10:14–23. 16321563 10.1016/j.tics.2005.11.006

[BHU282C40] Henson RNA 2003 Neuroimaging studies of priming. Prog Neurobiol. 70:53–81. 12927334 10.1016/s0301-0082(03)00086-8

[BHU282C41] Hirschfeld L Gelman S 1997 What young children think about the relationship between language variation and social difference. Cognitive Dev. 12:213–238.

[BHU282C42] Hurt HT Weaver CH 1972 Negro dialect, ethno-centricism, and the distortion of information in the communicative process. Cent States Speech J. 23:118–125.

[BHU282C43] Kinzler KD Dupoux E Spelke ES 2007 The native language of social cognition. Proc Natl Acad Sci USA. 104:12577–12580. 17640881 10.1073/pnas.0705345104 PMC1941511

[BHU282C44] Klasen M Kenworthy CA Mathiak KA Kircher TT Mathiak K 2011 Supramodal representation of emotions. J Neurosci. 31:13635–13643. 21940454 10.1523/JNEUROSCI.2833-11.2011 PMC6623280

[BHU282C45] Koelsch S Fritz T Von Cramon DY Muller K Friederici AD 2006 Investigating emotion with music: an fMRI study. Hum Brain Mapp. 27:239–250. 16078183 10.1002/hbm.20180 PMC6871371

[BHU282C46] Kouider S Dehaene S Jobert A Le Bihan D 2007 Cerebral bases of subliminal and supraliminal priming during reading. Cereb Cortex. 17:2019–2029. 17101688 10.1093/cercor/bhl110

[BHU282C47] Labov W 2006 The social stratification of English in New York City. 2nd ed. New York: Cambridge University Press.

[BHU282C48] Leitman DI Wolf DH Ragland JD Laukka P Loughead J Valdez JN Javitt DC Turetsky BI Gur GC 2010 “It's not what you say, but how you say it”: a reciprocal temporo-frontal network for affective prosody. Front Hum Neurosci. 4:4–19. 20204074 10.3389/fnhum.2010.00019 PMC2831710

[BHU282C49] Leopold DA O'Toole AJ Vetter T Blanz V 2001 Prototype-referenced shape encoding revealed by high-level after effects. Nat Neurosci. 4:89–94. 11135650 10.1038/82947

[BHU282C50] Lev-Ari S Keysar B 2010 Why don't we believe non-native speakers? The influence of accent on credibility. J Exp Soc Psychol. 46:1093–1096.

[BHU282C51] Lewis JW Talkington WJ Walker NA Spirou GA Jajosky A Frum C Brefczynski-Lewis JA 2009 Human cortical organization for processing vocalizations indicates representation of harmonic structure as a signal attribute. J Neurosci. 29:2283–2296. 19228981 10.1523/JNEUROSCI.4145-08.2009 PMC2774090

[BHU282C52] Mitchell RLC Elliott R Barry M Cruttenden A Woodruff PWR 2003 The neural response to emotional prosody, as revealed by functional magnetic resonance imaging. Neuropsychologia. 41:1410–1421. 12757912 10.1016/s0028-3932(03)00017-4

[BHU282C53] Morris JS Scott SK Dolan RJ 1999 Saying it with feeling: neural responses to emotional vocalizations. Neuropsychologia. 37:1155–1163. 10509837 10.1016/s0028-3932(99)00015-9

[BHU282C54] Mulac A Hanley TD Prigge DY 1974 Effects of phonolgical speech foreigness upon three dimensions of attitude of selected American listeners. Q J Speech. 60:411–420.

[BHU282C55] Müller NG Strumpf H Scholz M Baier B Melloni L 2013 Repetition suppression versus enhancement—it's quantity that matters. Cereb Cortex. 23:315–322. 22314047 10.1093/cercor/bhs009

[BHU282C56] Nakamura K Dehaene S Jobert A LeBihan D Kouider S 2007 Task-specific change o funconscious neural priming in the cerebral language network. Proc Natl Acad Sci USA. 104:19643–19648. 18042726 10.1073/pnas.0704487104 PMC2148342

[BHU282C57] Nonyane BAS Theobald CM 2007 Design sequences for sensory studies: achieving balance for carry-over and position effects. Br J Math Stat Psychol. 60:339–349. 17971273 10.1348/000711006X114568

[BHU282C58] Otten S Moskowitz GB 2000 Evidence for implicit evaluative in-group bias: affect-biased spontaneous trait inference in a minimal group paradigm. J Exp Soc Psychol. 36:77–89.

[BHU282C59] Pell M Leonard CL 2003 Processing emotional tine from speech in Parkinson's disease: a role for the basal ganglia. Cogn Affect Behav Neurosci. 3:275–288. 15040548 10.3758/cabn.3.4.275

[BHU282C60] Pelli DG 1997 The VideoToolbox software for visual psychophysics: transforming numbers into movies. Spat. Vis. 10:437–442. 9176953

[BHU282C61] Perrachione TK Chiao JY Wong PCM 2010 Asymmetric cultural effects on perceptual expertise underlie an own-race bias for voices. Cognition. 114:42–55. 19782970 10.1016/j.cognition.2009.08.012 PMC2784142

[BHU282C62] Philippon AC Cherryman J Bull R Vrij A 2007 Earwitness identification performance: the effect of language, target, deliberate strategies and indirect measures. Appl Cognit Psychol. 21:539–550.

[BHU282C63] Phillips ML Young AW Scott SK Calder AJ Andrew C Giampietro V Williams SC Bullmore ET Brammer M Gray JA 1998 Neural responses to facial and vocal expressions of fear and disgust. Proc R Soc B Biol Sci. 265:1809–1817. 10.1098/rspb.1998.0506 PMC1689379 9802236

[BHU282C64] Rorden C Karnath H-O Bonilha L 2007 Improving lesion–symptom mapping. J Cognit Neurosci. 19:1081–1088. 17583985 10.1162/jocn.2007.19.7.1081

[BHU282C65] Rosch E 1973 Natural categories. Cognit Psychol. 4:328–350.

[BHU282C66] Ryan EB Sebastian RJ 1980 The effects of speech style and social class background on social judgements of speakers. Br J Soc Clin Psychol. 19:229–233.

[BHU282C67] Sander D Grafman J Zalla T 2003 The human amygdala: an evolved system for relevance detection. Rev Neurosci. 14:303–316. 14640318 10.1515/revneuro.2003.14.4.303

[BHU282C68] Sander D Grandjean D Pourtois G Schwartz S Seghier ML Scherer KR Vuilleumier P 2005 Emotion and attention interactions in social cognition: brain regions involved in processing anger prosody. NeuroImage. 28:848–858. 16055351 10.1016/j.neuroimage.2005.06.023

[BHU282C69] Sander D Scheich H 2001 Auditory perception of laughing and crying activates the human amygdala regardless of attentional state. Cognit Brain Res. 12:181–198. 10.1016/s0926-6410(01)00045-3 11587889

[BHU282C70] Schirmer A Escoffier N Zysset S Koester D Striano T Friederici AD 2008 When vocal processing gets emotional: on the role of social orientation in relevance detection by the human amygdala. Neuroimage. 40:1402–1410. 18299209 10.1016/j.neuroimage.2008.01.018

[BHU282C71] Segaert K Weber K de Lange FP Petersson KM Hagoort P 2013 The suppression of repetition enhancement: a review of fMRI studies. Neuropsychologia. 51:59–66. 23159344 10.1016/j.neuropsychologia.2012.11.006

[BHU282C72] Stevenage SV Clarke G McNeill A 2012 The effect of regional accent on voice recognition. J Cogn Psychol. 24:647–653.

[BHU282C73] Tajfel H Billig M 1974 Familiarity and categorization in intergroup behavior. J Exp Soc Psychol. 10:159–170.

[BHU282C74] Tajfel H Billig MG Bundy RP Flament C 1971 Social categorization and intergroup behavior. Eur J Soc Psychol. 1:149–177.

[BHU282C75] Tajfel H Turner JC 1979 An integrative theory of inter-group conflict. In: Austin WG Wrochel S, editors. The social psychology of inter-group relations. Monterey, CA: Brooks/Cole.

[BHU282C76] Turk-Browne NB Yi DJ Leber AB Chun MM 2007 Visual quality determines the direction of neural repetition effects. Cereb Cortex. 17:425–433. 16565294 10.1093/cercor/bhj159

[BHU282C77] Turner JC 1975 Social comparison and social identity—some prospects for intergroup behavior. Eur J Soc Psychol. 5:5–34.

[BHU282C78] Turner JC Hogg MA Oakes PJ Reicher SD Wetherell MS 1987 Rediscovering the social group: a self-categorization theory. Oxford: Blackwell.

[BHU282C79] Valentine T 1991 A unified account of the effects of distinctiveness, inversion, and race in face recognition. Q J Exp Psychol. 43:161–204. 10.1080/14640749108400966 1866456

[BHU282C80] Van Bavel JJ Packer DJ Cunningham WA 2008 The neural substrates of in-group bias: a functional magnetic resonance imaging investigation. Psychol Sci. 19:1131–1139. 19076485 10.1111/j.1467-9280.2008.02214.x

[BHU282C81] Volz KG Kessler T von Cramon DY 2009 In-group as part of the self: in-group favoritism is mediated by medial prefrontal cortex activation. Soc Neurosci. 4:244–260. 19085561 10.1080/17470910802553565

[BHU282C82] Vuilleumier P 2005 How brains beware: neural mechanisms of emotional attention. Trends Cogn Sci. 9:585–594. 16289871 10.1016/j.tics.2005.10.011

[BHU282C83] Vuilleumier P Schwartz S Duhoux S Dolan RJ Driver J 2005 Selective attention modulates neural substrates of repetition printing and “implicit” visual memory: suppressions and enhancements revealed by fMRI. J Cognitive Neurosci. 17:1245–1260. 10.1162/0898929055002409 16197681

[BHU282C84] Whalen PJ 1998 Fear, vigilance, and ambiguity: Initial neuroimaging studies of the human amygdala. Curr Dir Psychol Sci. 7:177–188.

[BHU282C85] Wiethoff S Wildgruber D Grodd W Ethofer T 2009 Response and habituation of the amygdala during processing of emotional prosody. NeuroReport. 20:1356–1360. 19696688 10.1097/WNR.0b013e328330eb83

[BHU282C86] Wildgruber D Riecker A Hertrich I Erb M Grodd W Ethofer T Ackermann H 2005 Identification of emotional intonation evaluated by fMRI. Neuroimage. 24:1233–1241. 15670701 10.1016/j.neuroimage.2004.10.034

[BHU282C87] Williams A Garrett P Coupland N 1999 Dialect recognition. In: Preston DR, editor. Handbook of perceptual dialectology. Philadelphia: John Benjamins.

[BHU282C88] Witteman J Van Heuven VJP Schiller NO 2012 Hearing feelings: a quantitative meta-analysis on the neuroimaging literature of emotional prosody perception. Neuropsychologia. 50:2752–2763. 22841991 10.1016/j.neuropsychologia.2012.07.026

